# The Effect of Salt Reduction on the Microbial Community Structure and Metabolite Composition of Cheddar Cheese

**DOI:** 10.3390/foods13244184

**Published:** 2024-12-23

**Authors:** Xinping Wang, You Kang, Lei Gao, Yujuan Zhao, Yansong Gao, Ge Yang, Shengyu Li

**Affiliations:** 1 Department of Food Science and Engineering, College of Agricultural, Yanbian University, Yanji 133000, China; w18243399796@163.com; 2 Institute of Agro-Food Technology, Jilin Academy of Agricultural Sciences (Northeast Agricultural Research Center of China)/National R&D Center for Milk Processing, Changchun 130033, China; kangkang.1982@163.com (Y.K.); redhuman@126.com (L.G.); zhaojaas@163.com (Y.Z.); gysgerry@126.com (Y.G.); yangge1900@163.com (G.Y.)

**Keywords:** cheddar cheese, low salt, microorganism, metabolite

## Abstract

As consumer demand for low-salt diets increases, the development of low-salt cheese has emerged as a prevailing trend. To gain a deeper insight into the effects of salt reduction on cheddar cheese, this study used cheddar cheese with a 2.0% salt concentration (full salt, FS) as the standard control, exploring the differences in quality and composition between cheddar cheese with a 1.5% salt concentration (reduced salt, RS) and a 1.0% salt concentration (half salt, HS). The results revealed that, while the RS group exhibited significant differences in texture compared to the FS group, their physicochemical composition and microbial communities were similar, resulting in a product with quality comparable to traditional cheese. In contrast, the HS group differed notably from the FS group in terms of its physicochemical composition, texture, and microbial communities. Shifts in the microbial community within the HS group promoted enhanced protein metabolism, producing a substantial increase in free amino acids and volatile flavor compounds. In summary, cheddar cheese with a 1.5% salt concentration is similar to traditional varieties in terms of quality, while the 1.0% salt variety displays a more complex composition, due to microbial community shifts facilitating protein metabolism.

## 1. Introduction

Cheddar cheese is a hard, dry-salted cheese, one of the most popular and widely consumed cheeses globally. Before being transformed into cheese, raw milk undergoes multiple processing steps, including pasteurization, fermentation, coagulation, and maturation. During production, salt to achieve a 2.0% salt concentration is typically added to enhance the cheese’s flavor, improve the taste, and inhibit the growth of spoilage microorganisms, thereby extending its shelf life. However, long-term consumption of high-salt foods is known to pose health risks, leading to the development of cardiovascular and cerebrovascular diseases [1]. Consequently, the WHO recommends that adults restrict their daily salt consumption to less than 5 g [2], while the UK Department of Health and Social Care’s target for 2020 aimed to reduce the salt content in cheddar and hard cheeses to below 1.9 g per 100 g [3]. With growing global awareness of health risks, consumer demand for low-salt diets is increasing, impacting the production and consumption patterns of traditional high-salt foods, such as cheddar cheese.

To address this issue, various strategies have emerged in recent years to reduce the salt content in cheese. These include substituting NaCl with KCl or flavor additives [4], alongside techniques like ultrafiltration and the application of high hydrostatic pressure [5]. Among these approaches, direct salt reduction is the simplest and most cost-effective method. Previous studies have shown that salt reduction leads to enhanced microbial growth and excessive protein hydrolysis, both of which are key factors affecting the taste and texture of cheddar cheese [6]. In addition, some reports suggest that high-quality cheddar cheese can be produced with a low sodium level by controlling and monitoring protein hydrolysis and water activity [7]. Currently, research on low-salt cheese primarily focuses on product development, flavor improvement, and functional enhancement. However, studies on the dynamic changes in microbial communities and metabolic patterns of key flavor compounds in cheddar cheese, resulting from salt reduction, remain scarce. There is no comprehensive discussion on the relationship between microorganisms, metabolites, and salt concentration in cheddar cheese. Given cheese’s complex microbial diversity and metabolic mechanisms, variations in salt concentration can lead to significant shifts in the microbial community structure, which in turn affect flavor metabolites. Therefore, studying the relationship between salt concentration, cheese microorganisms, and metabolites is the key to solving the problem of unstable flavor quality, while maintaining the traditional flavor in low-salt cheese. From the unique perspective of the interaction between microorganisms and metabolites, this study explored the effect of the salt concentration on cheddar cheese, aiming to construct the relationship between salt concentration, microorganisms, and metabolites in cheese.

This study initially evaluated the effects of salt reduction on the physicochemical properties and texture of cheddar cheese. Subsequently, differences in volatile flavor compounds among cheddar cheese samples with varying salt concentrations were characterized using gas chromatography–mass spectrometry technology. Furthermore, 16S rRNA gene amplicon sequencing and non-target metabolomics technology were used to analyze the succession patterns of the microbial communities, differential changes in the metabolites, and the metabolic pathways. Ultimately, the relationship between microbial genera and differential metabolites was investigated. This study provides valuable insights into the impact of salt reduction on cheddar cheese, offering a scientific basis for improving the production of low-salt cheddar cheese.

## 2. Materials and Methods

### 2.1. Cheddar Cheese Manufacturing

After standardization, 18 kg of raw milk was evenly divided into three cheese vats (HYCV-407, manufactured by Jilin Wanzhi Technology Co., Ltd., Changchun, China). It was then pasteurized at 65 °C for 30 min and cooled down to 37 °C. After allowing the starter culture (R707, Chr. Hansen, Denmark) to work for 60 min, 0.18 g CaCl_2_ (0.1 g/L) and 0.24 g rennet (CHY-MAX Power NB, 0.014 g/L, Chr. Hansen, Denmark) were added to the pasteurized milk, resulting in coagulation within approximately 45 min at 37 °C. The curd was divided into 2 cm cubes using a cheese knife and gently stirred while being heated to 42 °C, with a gradual increase in temperature of no more than 2 °C every 5 min, until the pH level reached was 6.1–6.2. Subsequently, the whey was separated, and the curd was stirred at 38 °C, with a rotation every 15 min, until the pH level dropped to 5.4–5.5. The curd was milled and salted (NaCl) to achieve a salt concentration of 2.0% (full salt, FS), 1.5% (reduced salt, RS), and 1.0% (half salt, HS) (*w*/*w*). It was then placed in a round cheese mold, with a diameter of 12.8 cm, and pressed at a pressure of 0.2 MPa for 1.5 h, followed by pressing at 0.5 MPa for 20 h. After pressing, the cheddar cheese block was vacuum packed using food-grade plastic sealing bags and matured for 90 d at 8 °C and 70% humidity conditions before sampling; each sample underwent three independent replicate experiments [8].

### 2.2. Determination of Physicochemical Composition

The protein, fat, and moisture content were determined using the reference methods, according to Bradley et al. [9]. And the protein and fat content of the cheeses were measured according to the Kjeldahl method and Babcock method, respectively. The moisture content of each cheddar sample was determined using a gravimetric method, by heating them to a constant weight at 102 °C. The salt content was determined according to the Mohr method [10], in which chlorides are titrated with a silver nitrate solution in the presence of chromate anions. The pH level of the cheese samples was determined using a digital pH meter (Testo205, Testo Ltd., Titisee-Neustadt, Germany).

### 2.3. Determination of Texture Profile

The texture profile analysis of the cheese was conducted using a standardized texture analyzer (Stable Micro Systems Ltd., Godalming, UK). Prior to testing, a cheese specimen with dimensions of 1.5 cm × 1.5 cm × 1.5 cm was conditioned at room temperature (25 °C) for 2 h. The analysis was performed utilizing a P/5 probe and the parameters were set in accordance with the methodology outlined in the work by Wang et al. [11].

### 2.4. Determination of Volatile Flavor Compound

The volatile flavor compounds were quantitatively analyzed using headspace solid-phase microextraction, coupled with gas chromatography–mass spectrometry (HS-SPME/GC-MS) (5977B GC/MSD, Agilent Technologies Co., Santa Clara, CA, USA). Specifically, a 75 µm DVB/CAR/PDMS fiber (Supelco, Bellefonte, PA, USA) was utilized for solid-phase microextraction to capture the volatile compounds present in the headspace. Following a 40 min equilibrium period at 60 °C in a water bath, the extracted volatile compounds were then analyzed by the GC-MS system, which was equipped with a GCMS-QP2010 gas chromatograph mass spectrometer (Shimadzu Corp., Kyoto, Japan). The system utilized a DB-Wax column (30 m × 250 μm × 0.25 μm). The initial temperature was kept at 40 °C for 4 min, then increased to 245 °C at a rate of 5 °C min^−1^, and kept at that temperature for 5 min. A preliminary identification of the compounds was accomplished through a comparative analysis of their fragmentation signatures, using electron ionization mass spectrometry, against those recorded in the National Institute of Standards and Technology database. Further confirmation of these identifications was achieved by matching their linear retention indices. Finally, the relative concentrations of the detected volatile compounds were determined using the relative peak area method.

### 2.5. Determination of Microbial

Total genomic DNA samples were extracted using the OMEGA Soil DNA Kit (M5635-02) (Omega Bio-Tek, Norcross, GA, USA), following the manufacturer’s instructions. PCR amplification of the bacterial 16S rRNA genes V3–V4 region was performed using the forward primer 338F (5′-ACTCCTACGGGAGGCAGCA-3′) and the reverse primer 806R (5′-GGACTACHVGGGTWTCTAAT-3′). Seven base pair sample-specific barcodes were incorporated into the primers to facilitate multiplex sequencing. The PCR components contained 5 μL of buffer (5×), 0.25 μL of Fast Pfu DNA Polymerase (5 U/μL), 2 μL (2.5 mM) of dNTPs, 1 μL (10 μM) of each Forward and Reverse primer, 1 μL of DNA Template, and 14.75 μL of ddH_2_O. The thermal cycling protocol involved an initial denaturation step at 98 °C for 5 min, followed by 25 cycles of denaturation at 98 °C for 30 s, annealing at 53 °C for 30 s, and extension at 72 °C for 45 s, with a final extension step at 72 °C for 5 min. The PCR amplicons were purified with Vazyme VAHTSTM DNA Clean Beads (Vazyme, Nanjing, China), and the resulting products underwent individual quantification and quality assessment using the Illumina MiSeq sequencing platform, provided by Illumina Inc. (San Diego, CA, USA). The high-quality sequences, exhibiting a homology threshold of 97%, were subsequently classified into operational taxonomic units (OTUs) for further analysis.

### 2.6. Determination of Metabolite

The ultra-high performance liquid chromatography (UHPLC) system (Vanquish, Thermo Fisher Scientific, Waltham, MA, USA), coupled with a mass spectrometer (Orbitrap Exploris 120, Thermo Fisher Scientific, Waltham, MA, USA), was employed for the metabolite analysis of the cheese. This method is based on the work by Wang et al. [12], with slight modifications. A total of 50 mg of the sample was accurately weighed and placed into a 2 mL centrifuge tube, followed by the addition of 200 µL of methanol and 200 µL of methyl tert-butyl ether solution. The mixture was vortexed for 1 min and then centrifuged at 12,000 rpm for 15 min at 4 °C. The supernatant was filtered through a 0.22 µm membrane and transferred into a detection vial for liquid chromatography–mass spectrometry (LC-MS) analysis.

The UHPLC system was utilized for liquid chromatography analysis, with the mobile phase consisting of formic acid in acetonitrile and formic acid in water for the positive ion mode, and acetonitrile and ammonium formate for the negative ion mode. The mass spectrometer, equipped with an electrospray ionization (ESI) source, employed MS1 and MS/MS (Full MS-ddMS2 mode, data-dependent MS/MS) for mass spectral detection. The ESI (+) and ESI (−) were set at 3.50 kV and −2.50 kV, respectively, with a resolution of 60,000 full width at half maximum (FWHM). For the secondary fragmentation of the top four ions, higher energy collisional dissociation was employed, with a normalized collision energy of 30% and a resolution of 15,000 FWHM. For further processing, the raw data were imported into the metabolomics processing software, Progenesis QI, www.nonlinear.com (Waters Corporation, Milford, CT, USA).

### 2.7. Statistical Analysis

The experimental data are presented as mean values, with the corresponding standard errors, to provide a comprehensive understanding of the variations in the results. To identify significant variations in the physicochemical composition and texture, the SPSS 27.0 software package (IBM Corp., Armonk, NY, USA) was utilized. The independent sample *t*-test and two-way ANOVA statistical methods were employed to determine whether there were statistically significant differences between the examined variables. A threshold of *p* < 0.05 was used to determine statistical significance, meaning that any differences below this value were considered noteworthy. Additionally, to assess the potential relationship between the metabolites and microorganisms present in the cheese samples, Pearson’s rank correlation analysis was conducted, providing insights into the correlation between the microflora and their associated metabolic products.

## 3. Results and Discussion

### 3.1. Physicochemical Composition Analysis

The physicochemical composition of three types of cheddar cheese with varying salt concentrations is shown in Table 1. The FS group exhibited significant differences in regard to all its physicochemical parameters compared to the HS group, while no significant differences were found in the protein content, fat content, and MNFS between the FS and RS groups. Consequently, the physicochemical composition of the FS and RS groups is relatively similar, except for the significant differences in salt and salt in moisture. As the salt content decreases, the concentrations of protein, fat, and salt decrease in cheese, while the S/M ratio, FDM, and pH value also decrease, and the moisture content and MNFS increase. With the exception of FDM, the correlations between other aspects of the physicochemical composition of the cheeses and the salt addition amount are consistent with a previous study [6]. The reason for the different correlations may be due to a smaller increase in the water content or a more substantial decrease in the fat content resulting from the salt reduction in this study.

### 3.2. Texture Profile Analysis

The texture profile analyses of the three types of cheddar cheese with varying salt concentrations are shown in Table 2. As the salt content decreases, the hardness and chewiness of the cheese decrease significantly, which is consistent with previous research, indicating that the hardness of cheese decreases with the decrease in salt content [7] and chewiness is often positively correlated with hardness [13]. The decrease in salt content leads to increased moisture in the cheese, which dilutes the protein concentration and weakens the protein interactions. Consequently, the cheese becomes more deformable and easier to chew [14]. Conversely, the elasticity and adhesiveness values of cheddar cheese increased with the reduction in salt content. This is likely due to the increased moisture, which causes casein proteins to absorb water and swell, thereby enhancing the cheese’s elasticity. Concurrently, the reduction in salt content facilitates the breakdown of proteins and fats into smaller molecules, increasing the viscosity of the cheese [15]. Therefore, reducing the salt content in cheese may lead to significant changes in the texture by affecting the moisture content.

### 3.3. Volatile Flavor Compound Analysis

**Figure 1:**
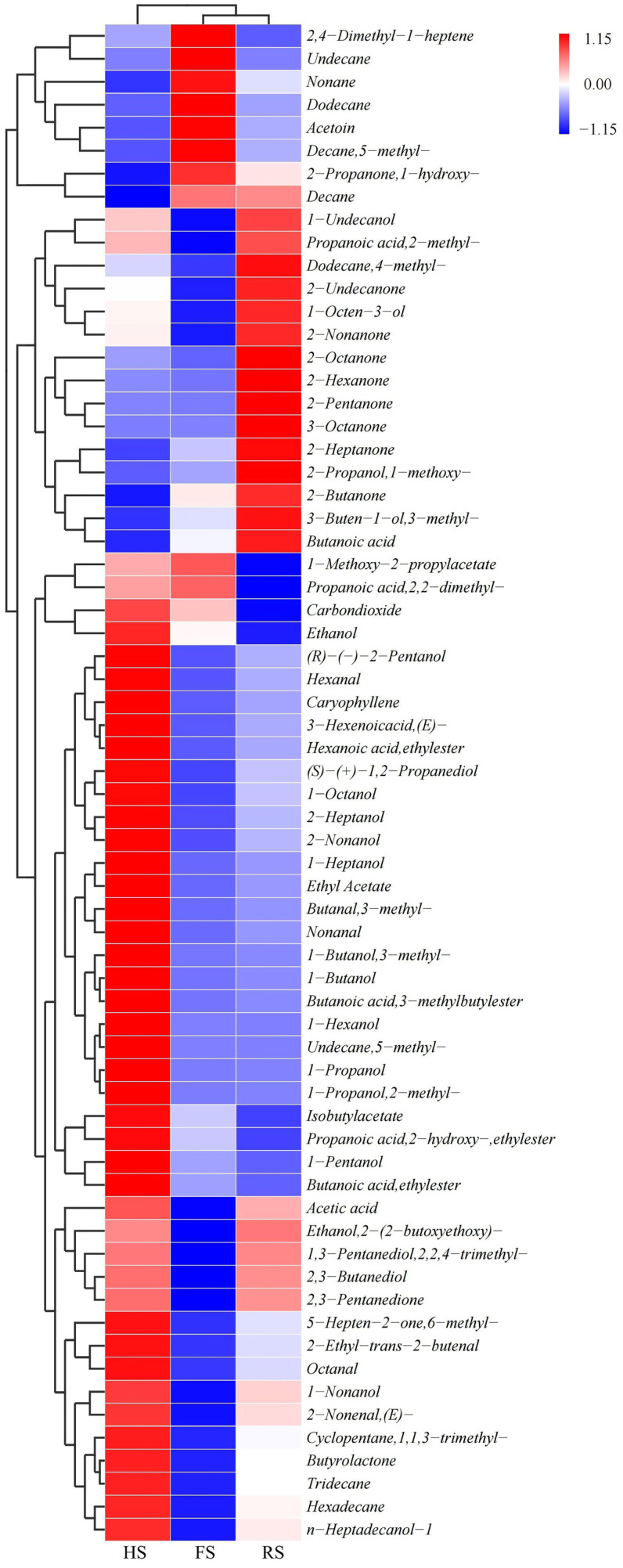
Cluster heatmap of differential volatile flavor compounds in three types of cheddar cheese with varying salt concentrations. The color scale reflects the concentration of volatile flavor compounds, with red indicating a high concentration and blue indicating a low concentration.

In this study, a total of 66 volatile flavor compounds were identified with significant differences between the groups, and the cluster analysis results are shown in Figure 1. These compounds include 22 alcohols, 11 ketones, 7 esters, 5 aldehydes, 5 acids, and 16 other compounds. The HS group exhibited the highest total concentration of volatile compounds, with the highest content of acids, esters, alcohols, and aldehydes, while the RS group had the highest ketone content. The results of the cluster analysis indicated that the RS group and the FS group shared a higher degree of similarity.

Reduced-salt cheese exhibits higher concentrations of volatile compounds, due to the early growth of bacteria and increased enzymatic activity [7]. The HS group exhibited significantly higher levels of acids, including acetic acid, a primary organic acid in cheddar cheese [16]. Acetic acid can be synthesized through the catabolic metabolism of lactose, citrate, and free fatty acids, or via amino acid metabolism, and is directly associated with a sour taste [17]. The highest concentration of acetic acid in the HS group may indicate that salt reduction enhances the sour taste of cheese samples. The HS group also had significantly higher levels of esters, particularly ethyl caproate and ethyl butanoate, consistent with previous research findings [7]. Ethyl acetate, known for its fruity flavor, and ethyl butanoate, with its pineapple-like aroma, are formed through the esterification of ethanol and acids or amino acid metabolism. While present at low concentrations, they play a significant role in the overall sensory experience of cheese [18]. The HS group also had notably higher levels of alcohols, including ethanol, which accounted for the highest proportion, aligning with the findings by Dugat-Bony et al. [19]. The HS group exhibited significantly higher levels of aldehydes, which are primarily derived from amino acid and fatty acid metabolism in cheese. Branched aldehydes and aldehydes derived from amino acids are often positively correlated with cheese flavor. For instance, hexanal, produced through β-oxidation of fatty acids, imparts a green flavor, while 3-methylbutanal, formed via amino acid metabolism, has a fruity aroma [20]. The RS group, in contrast, had notably higher levels of ketones, particularly 2-nonanone, which is formed through β-oxidation and decarboxylation of fatty acids, contributing a hot milk flavor [21]. This contrasts with the findings by Dugat-Bony et al. [19], who discovered fewer ketones in low-salt (1.3%) cheese. This discrepancy may be attributed to differences in the pH value, water activity, and non-fermenting lactic acid bacteria, which affect the activity of cheese lipase [22]. Therefore, the reduction in salt content possibly leads to the intensification of amino acid and fatty acid metabolism, resulting in an increase in the complexity of volatile flavor compounds in cheeses.

### 3.4. Microbial Analysis

**Figure 2:**
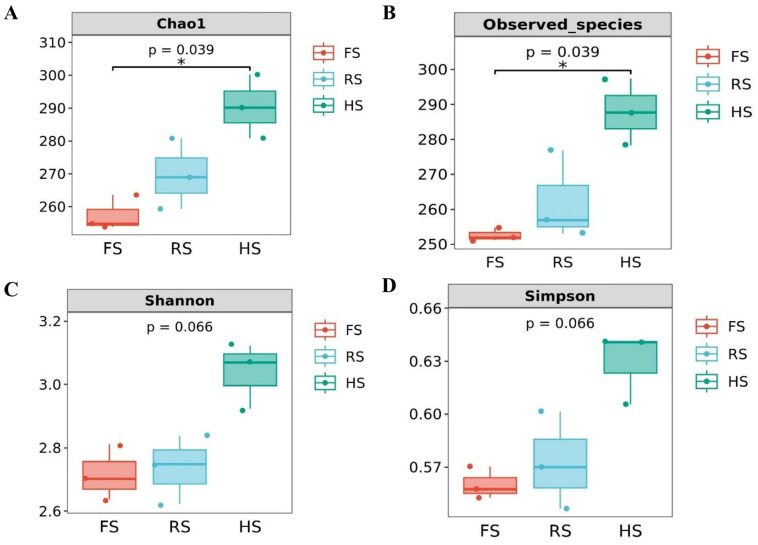
Alpha diversity analysis of three types of cheddar cheese with varying salt concentrations: Chao1 index (**A**); observed species index (**B**); Shannon index (**C**); and Simpson index (**D**) (* *p* < 0.05).

**Figure 3:**
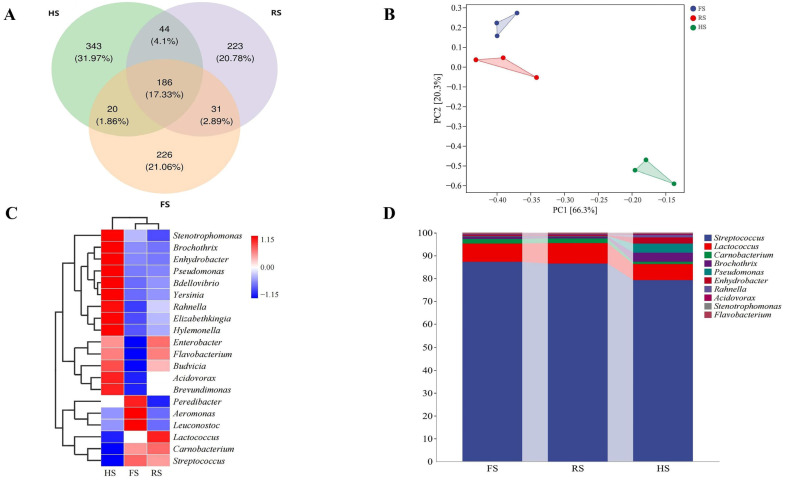
Changes in microbial communities in three types of cheddar cheese with varying salt concentrations: Chao1 index OTU Venn diagram (**A**); principal coordinate analysis (PCoA) of all the samples by the weighted UniFrac distance (**B**); cluster heatmap at the genus level (**C**); relative abundance at the genus level (**D**).

This study used 16S rRNA gene amplicon sequencing to elucidate the effects of salt reduction on microbial diversity and abundance in cheddar cheese (Figure 2). As the salt content decreases, the Chao1, observed species, Shannon, and Simpson indices exhibit an upward trend (Figure 2A–D), indicating a gradual increase in both microbial diversity and abundance within the three cheese groups. This is due to the reduction in osmotic pressure as the salt concentration decreases, which enables microorganisms that thrive in low-salt environments to survive better and proliferate, thereby enhancing the diversity in and abundance of the microbial community [23]. The Venn diagram displays the OTU clustering in cheddar cheese (Figure 3A). Notably, the HS group exhibits the highest number of OTUs, accounting for 55.26% of the total. This suggests a high level of microbial diversity, supporting the conclusion that reduced salt content increases microbial diversity. Meanwhile, the PCoA analysis showed that the FS group and RS group were relatively close in distance, indicating their high similarity (Figure 3B).

The genus-level heatmap (Figure 3C) and microbial taxonomy diagram (Figure 3D) revealed *Streptococcus* and *Lactobacillus* as the primary bacterial genera. Notably, there was no statistically significant difference between the FS group and the RS group, which aligns with the findings by Dugat-Bony et al. [19], who reported that a 20% reduction in sodium chloride content did not significantly alter the microbial composition of the cheese. However, in the HS group, a significant decrease in the abundance of *Streptococcus*, *Lactobacillus*, and *Carnobacterium* was observed, accompanied by a notable increase in the abundance of *Brochothrix*, *Pseudomonas*, and *Enterobacter*. Dugat-Bony et al. [23] observed that reducing the salt content inhibits the growth of *Debaryomyces hansenii* yeast in cheese, but simultaneously enhances the growth of *Pseudomonas* species. Tabla et al. [24] reported that after 30 d of cheese ripening, only *Pseudomonas* species continued to be inhibited by salt, attributed to its relatively poor salt tolerance within the cheese environment. *Pseudomonas* is an important spoilage bacterium in cheese, negatively impacting its sensory characteristics. For instance, certain strains belonging to this genus are capable of producing pigments [25], biogenic amines [26], and various volatile compounds [27] during the ripening process, leading to undesirable alterations in the cheese’s quality and flavor. Similarly, *Enterobacter* poses a potential contamination risk, contributing to spoilage. While *Carnobacterium* and *Brochothrix* are non-lactic acid bacteria involved in cheese production, their roles are still not well understood. *Streptococcus* and *Lactobacillus*, which are common microorganisms found in cheese, possess the function of enhancing the aroma characteristics of the cheese. In conclusion, meticulous control of the salt content in cheese is crucial for regulating the microbial community structure. While a proper reduction in salt content will not significantly alter this structure, excessive reduction can shift the balance towards unfavorable microbial genera, thereby posing a risk of harmful microorganism proliferation. These findings offer valuable insights into strategies for regulating microbial community composition to enhance the shelf life of cheese.

### 3.5. Metabolite Analysis

**Figure 4:**
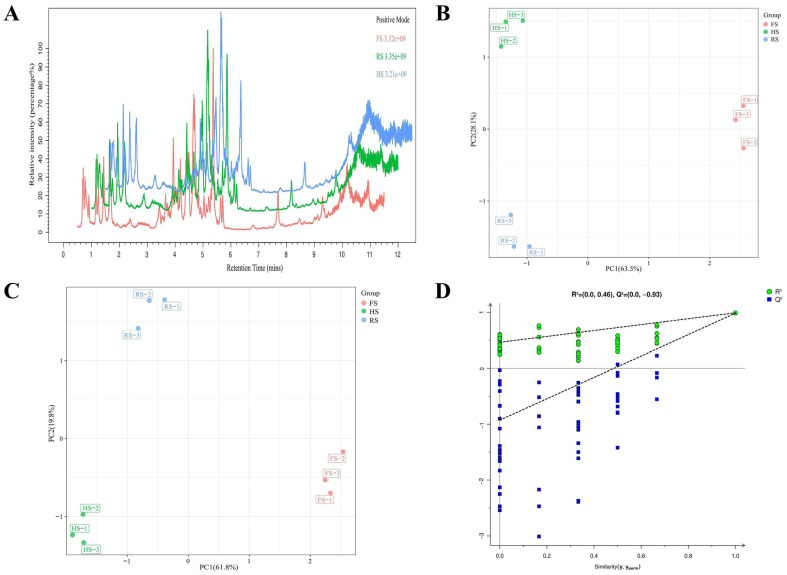
Metabolomic analysis of three types of cheddar cheese with varying salt concentrations: total ion chromatogram (**A**); PCA score plot (**B**); PLS-DA score plot (**C**); PLS-DA permutation test plot (**D**).

A total of 23,545 substance peaks were detected using metabolomics methods for subsequent comparative analysis of the differential metabolites (Figure 4A). The unsupervised PCA model and supervised PLS-DA model were used to distinguish the differences in the metabolites between the samples. Among them, the cumulative variance contribution rate of PC1 (63.5%) and PC2 (28.1%) was 91.6% (Figure 4B), indicating sufficient sample information [28]. The PLS-DA score plot demonstrated inter-group differences and intra-group consistency (R^2^X = 0.916, R^2^Y = 0.990, Q^2^ = 0.983) (Figure 4C). The results of the permutation test [R^2^ = (0.0, 0.46), Q^2^ = (0.0, −0.93)] (Figure 4D) indicate that the model in this study has good predictability and reproducibility, without overfitting.

**Figure 5:**
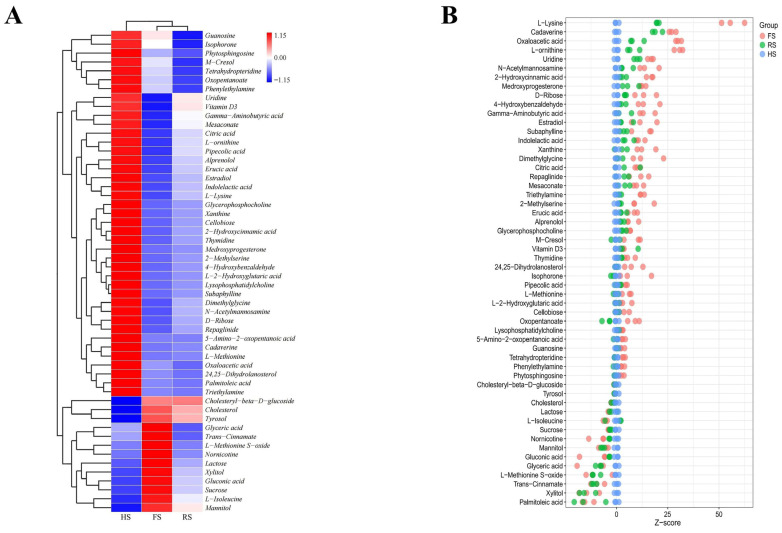
Changes in the metabolites in cheddar cheese with three different salt concentrations: cluster heatmap of differential metabolites (**A**); Z-score plot of differential metabolites (**B**).

Based on different levels of importance (VIP ≥ 1) and *p*-values (*p* < 0.05), a total of 54 differential metabolites were screened and were subsequently clustered into a heatmap (Figure 5A) and a Z-score plot (Figure 5B), to summarize the changes in the metabolites present in cheese with varying salt concentrations. These metabolites primarily consist of amino acids, fatty acids, carbohydrates, and organic acids, as well as their respective derivatives. From a holistic perspective, amino acids and their derivatives, including gamma-aminobutyric acid, L-lysine, and L-ornithine, are more abundant in the HS group, enhancing the flavor of the cheese. The HS group exhibits a higher abundance of fatty acids and derivatives, including lysophosphatidylcholine, palmitoleic acid, and oxopentanoate. The accumulation of lysophosphatidylcholine may stem from phospholipase-catalyzed phospholipid hydrolysis caused by *Pseudomonas* [29]. Conversely, the abundance of carbohydrates, specifically sucrose and lactose, is significantly lower in the HS group. This observation may correlate with the higher abundance and diversity of the microbial communities in low-salt environments, which promote carbohydrate consumption, subsequently generating metabolites like alcohols and acids [30]. Regarding organic acids and their derivatives, the concentrations of oxaloacetic acid, erucic acid, and citric acid are higher in the HS group, which aligns with previous findings that low-salt treatment increases organic acids [31]. At an individual level, notable elevations (Z-score > 25) in L-lysine, cadaverine, oxaloacetic acid, and L-ornithine in the HS group suggest that a low-salt environment promotes an increase in these specific metabolites.

**Figure 6:**
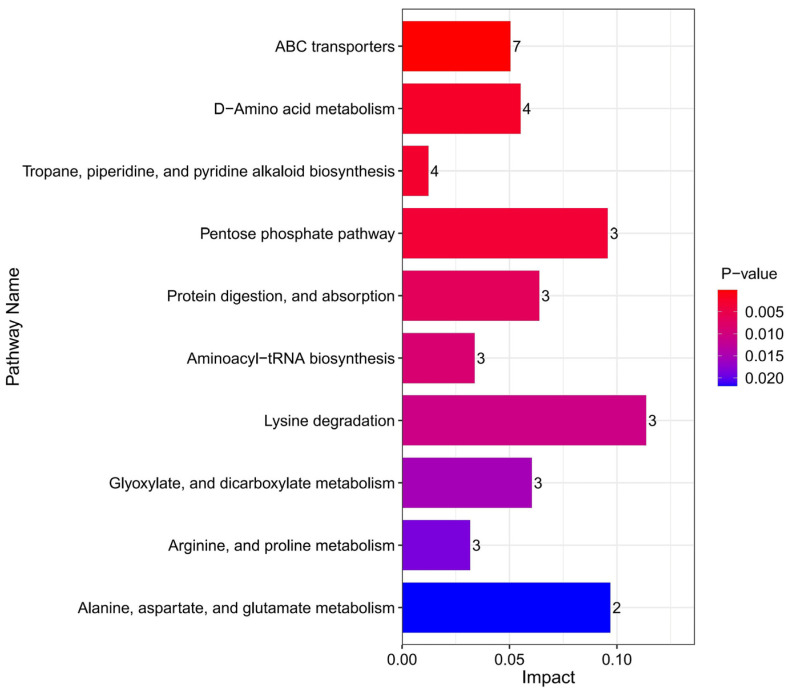
Metabolic pathway enrichment bar chart for cheddar cheese. The number represents the quantity of metabolites involved, with a redder color indicating a smaller *p*-value and a bluer color indicating a larger *p*-value.

**Figure 7:**
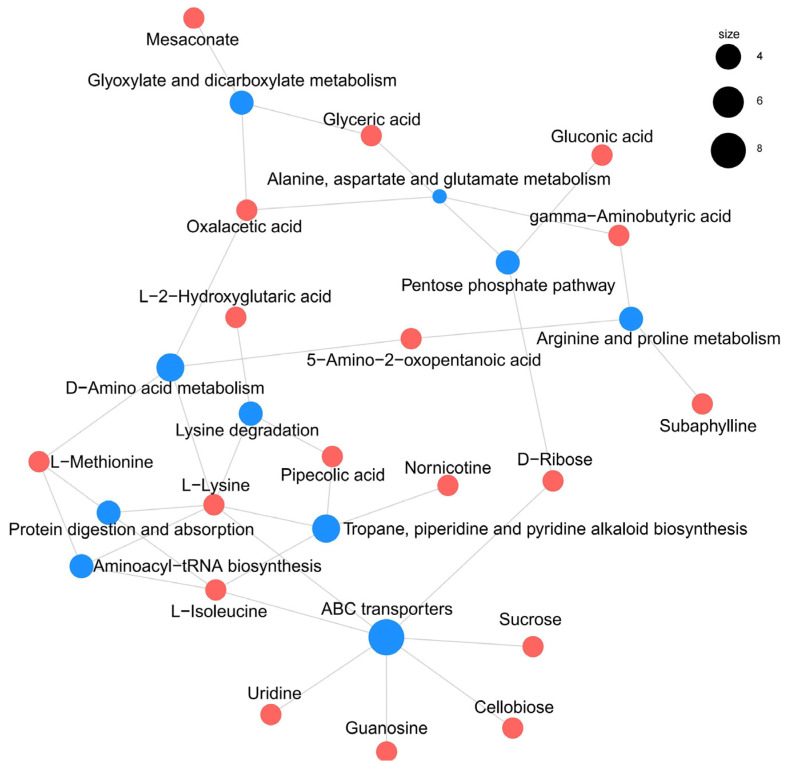
Metabolic pathway association network diagram for cheddar cheese. The blue dots represent metabolic pathways, while the red dots represent metabolites. The size of the blue dot indicates the number of metabolites associated with it.

The analysis of metabolite pathways can further elucidate the inter-group differences [32]. Based on the Kyoto Encyclopedia of Genes and Genomes (KEGG) database for enrichment analysis [33], and a comprehensive consideration of the *p*-values and impact values, the ten most significant metabolic pathways were selected (Figure 6), most of which are related to amino acid metabolism. This suggests that amino acid metabolism appears to be the main pathway responsible for metabolic differences in cheese caused by salt reduction. Integrated analysis of the pathways reveals a metabolic network diagram (Figure 7), where the same metabolite participates in multiple pathways simultaneously, indicating its significant impact. L-lysine has been identified as a key metabolite involved in six pathways. In recent years, the metabolic pathways of fermented foods have gradually attracted increasing attention. Li et al. [34] used metabolomics to analyze low-salt chili sauce and found that amino acid metabolism is the main pathway for flavor formation. Similarly, Zhao et al. [35] analyzed the metabolic process of low-salt Chinese cabbage using the KEGG database and revealed that the biosynthesis of aminoacyl tRNA, along with the metabolism of glycine, serine, and threonine, significantly impacts differential metabolites. These studies, along with the findings from this research, indicate that the metabolic changes induced by salt reduction in fermented foods are intimately linked to amino acid metabolism. Since amino acid metabolism produces a range of flavor compounds, including aldehydes, esters, acids, and alcohols, this perhaps explains the unique flavor profile of low-salt fermented foods.

### 3.6. Correlation Analysis

**Figure 8:**
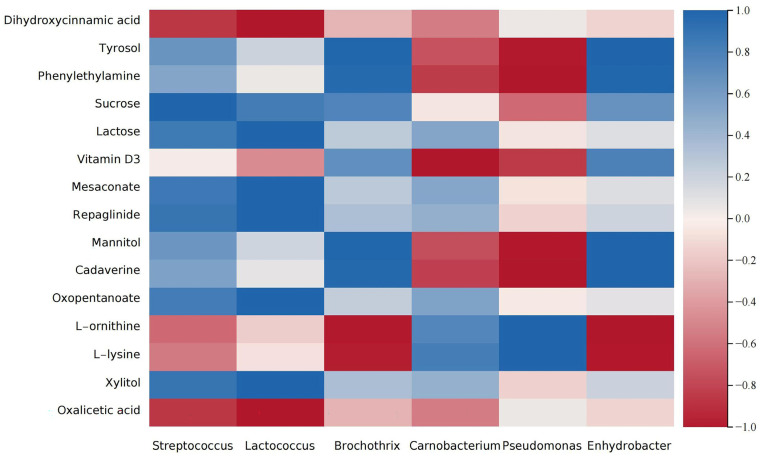
Correlation heatmap for the major microbial genera and key differential metabolites in three groups of cheddar cheese. The horizontal axis represents microbial genera, while the vertical axis represents metabolites. The intensity of the correlation is indicated by the color and saturation, with red representing a positive correlation and blue representing a negative correlation.

Microbial metabolic activities play a pivotal role in the formation of metabolites in cheese. Consequently, we selected the top six most abundant bacterial genera in cheese and conducted Pearson’s correlation analysis of the metabolites (*p* < 0.05, |R| ≥ 0.6) (Figure 8). The results revealed that *Streptococcus* and *Lactococcus* exhibited positive correlations with organic acids and negative correlations with lactose and other carbohydrates. This is attributed to the abundant lactate dehydrogenase present in these bacteria, which enables them to decompose lactose and other carbohydrates, leading to the production of organic acids [36]. In addition, the correlation patterns between *Streptococcus* and *Lactococcus* with various metabolites are similar, indicating that they have similar metabolic pathways, which indirectly supports their synergistic relationship [37]. In contrast, *Brochothrix* and *Enhydrobacter* displayed positive correlations with amino acids and negative correlations with lactose and other carbohydrates, indicating their potential capacity to degrade macromolecules, such as proteins and lactose and other carbohydrates in cheese. Conversely, *Carnobacterium* showed positive correlations with lactose and other carbohydrates and negative correlations with amino acids. This opposite correlation pattern compared to Brochothrix and Enhydrobacter may explain the divergent trends observed when the salt content is reduced. Additionally, *Pseudomonas* was positively correlated with biogenic amines and negatively correlated with amino acids, indicating that *Pseudomonas* facilitate the decarboxylation of amino acids, leading to the production of biogenic amines. This conclusion is in line with the report by Marcobal et al. [38], which states that *Pseudomonas* fluorescens can convert lysine into cadaverine. In summary, the unique metabolites formed and altered in cheese are the cumulative result of the intricate interplay between various microorganisms.

## 4. Conclusions

This study elucidates the effects of salt reduction on cheddar cheese. Compared to traditional cheddar cheese, the varieties with a 1.5% salt concentration exhibit alterations in regard to certain aspects of their physicochemical composition and texture, but their microbial community structure is not altered significantly, thereby preserving volatile flavor compounds similar to those of traditional varieties. However, the 1.0% salt concentration varieties exhibited significant changes in their physicochemical composition, texture, and microbial community structure. Specifically, a significant decrease in the abundance of *Streptococcus*, *Lactobacillus*, and *Carnobacterium* was observed, and this was accompanied by a notable increase in the abundance of *Brochothrix*, *Pseudomonas*, and *Enterobacter*. These microbial shifts stimulate protein metabolism and amino acid accumulation within the cheese, leading to an increase in the complexity of volatile flavor compounds, while there is a risk that harmful microorganisms may pose a threat to food safety. Amino acid metabolism is the primary pathway for generating differential metabolites, with L-lysine serving as a pivotal metabolite. In summary, cheddar cheese with a 1.5% salt concentration effectively preserves the traditional features of the cheese, while reducing the salt content, providing a scientific foundation for the development of healthier cheese products. Although cheddar cheese with a 1.0% salt concentration may deviate from the classic flavor profile, it pioneers a new avenue for creating flavor-diverse low-salt cheese products, by harnessing the innovative potential of microbe-mediated metabolic pathways.

## Figures and Tables

**Table 1:** Physicochemical composition of cheddar cheese.

Sample	Protein (%)	Fat (%)	Moisture (%)	Salt (%)	FDM (%)	MNFS (%)	S/M (%)	pH
FS	24.58 ± 0.31 ^a^	33.65 ± 0.56 ^a^	35.13 ± 0.13 ^c^	1.57 ± 0.02 ^a^	51.84 ± 0.11 ^a^	52.95 ± 0.64 ^b^	4.47 ± 0.11 ^a^	5.26 ± 0.01 ^a^
RS	24.43 ± 0.42 ^a^	32.79 ± 0.42 ^ab^	36.28 ± 0.06 ^b^	1.21 ± 0.03 ^b^	51.46 ± 0.07 ^b^	53.98 ± 0.43 ^b^	3.34 ± 0.08 ^b^	5.12 ± 0.02 ^b^
HS	23.42 ± 0.53 ^b^	31.87 ± 0.83 ^b^	38.03 ± 0.09 ^a^	0.83 ± 0.01 ^c^	51.43 ± 0.13 ^b^	55.82 ± 0.81 ^a^	2.18 ± 0.03 ^c^	4.98 ± 0.01 ^c^

MNFS: moisture in non-fat substance; FDM: fat in dry matter; S/M: salt in moisture. Values in columns marked with different lowercase letters are significantly different (*p* < 0.05).

**Table 2:** Texture profile analysis of cheddar cheese.

Sample	Hardness (g)	Elasticity	Chewiness	Adhesiveness
FS	2330.73 ± 5.33 ^a^	0.96 ± 0.03 ^b^	1853.79 ± 2.26 ^a^	1292.38 ± 3.07 ^c^
RS	2150.73 ± 4.53 ^b^	1.01 ± 0.02 ^b^	1592.71 ± 2.43 ^b^	1502.38 ± 2.03 ^b^
HS	1880.51 ± 3.02 ^c^	1.17 ± 0.04 ^a^	1385.52 ± 2.39 ^c^	1713.16 ± 2.99 ^a^

Values in columns marked with different lowercase letters are significantly different (*p* < 0.05).

## Data Availability

The original contributions presented in this study are included in the article. Further inquiries can be directed to the corresponding author.
